# Supplementary material for: How Volatilities Nonlocal in Time Affect the Price Dynamics in Complex Financial Systems
Source: PLoS One. 2015 Feb 27;10(2):e0118399. doi: 10.1371/journal.pone.0118399 (PMC4344208; doi:10.1371/journal.pone.0118399)
Supplement: S1 Appendix — (PDF) [file pone.0118399.s001.pdf]

S1 Appendix

Calculation for $P^{+}(t)|_{\Delta v(t')>0}$, $P^{+}(t)|_{\Delta v(t')<0}$ and $P_0(t)$, and relation among them

We present the calculation for $P^{+}(t)|_{\Delta v(t')>0}$, $P^{+}(t)|_{\Delta v(t')<0}$ and $P_0(t)$, and derive the relation among them.

Calculation for $P^{+}(t)|_{\Delta v(t')>0}$, $P^{+}(t)|_{\Delta v(t')<0}$ and $P_0(t)$

The length of the time series is denoted by T. According to the definition of $\Delta v(t')$, T_2 is the long time window. In the calculation, t' should satisfy $t' - T_2 \geq 0$ and $t' + t \leq T$. With t' ranging from T_2 to $T - t$, we count the number of the following cases:

(a) $\Delta v(t') > 0$ and $r(t' + t) > 0$;
(b) $\Delta v(t') > 0$ and $r(t' + t) < 0$;
(c) $\Delta v(t') < 0$ and $r(t' + t) > 0$;
(d) $\Delta v(t') < 0$ and $r(t' + t) < 0$,

and denote them by $n^{r+}_{\Delta v+}$, $n^{r-}_{\Delta v+}$, $n^{r+}_{\Delta v-}$ and $n^{r-}_{\Delta v-}$, respectively.

From the definition of the normalized logarithmic return, $r(t' + t)$ and $\Delta v(t')$ are almost impossible to be zero. Having examined the data, we confirm that there are no data points with $\Delta v(t') = 0$ or $r(t' + t) = 0$ in the time series we analyze.

Then the probability of $r\,(t' + t) > 0$ on the condition of $\Delta v(t') > 0$ is

$$P^{+}(t)|_{\Delta v(t')>0} = \frac{n^{r+}_{\Delta v+}}{n^{r+}_{\Delta v+} + n^{r-}_{\Delta v+}},$$

and the probability of $r\,(t' + t) > 0$ on the condition of $\Delta v(t') < 0$ is

$$P^{+}(t)|_{\Delta v(t')<0} = \frac{n^{r+}_{\Delta v-}}{n^{r+}_{\Delta v-} + n^{r-}_{\Delta v-}}.$$

The unconditional probability $P_0(t)$ is

$$P_0(t) = \frac{n^{r+}_{\Delta v+} + n^{r+}_{\Delta v-}}{n^{r+}_{\Delta v+} + n^{r+}_{\Delta v-} + n^{r-}_{\Delta v+} + n^{r-}_{\Delta v-}}.$$

Relation among $\boldsymbol{P^{+}(t)|_{\Delta v(t')>0}}$, $\boldsymbol{P^{+}(t)|_{\Delta v(t')<0}}$ and $\boldsymbol{P_0(t)}$

$$P^{+}(t)|_{\Delta v(t')>0} - P_0(t) = \frac{n^{r+}_{\Delta v+} n^{r-}_{\Delta v-} - n^{r+}_{\Delta v-} n^{r-}_{\Delta v+}}{(n^{r+}_{\Delta v+} + n^{r-}_{\Delta v+})(n^{r+}_{\Delta v+} + n^{r+}_{\Delta v-} + n^{r-}_{\Delta v+} + n^{r-}_{\Delta v-})}.$$

$$P^{+}(t)|_{\Delta v(t')<0} - P_0(t) = \frac{n^{r+}_{\Delta v-} n^{r-}_{\Delta v+} - n^{r+}_{\Delta v+} n^{r-}_{\Delta v-}}{(n^{r+}_{\Delta v-} + n^{r-}_{\Delta v-})(n^{r+}_{\Delta v+} + n^{r+}_{\Delta v-} + n^{r-}_{\Delta v+} + n^{r-}_{\Delta v-})}.$$

If $P^{+}(t)|_{\Delta v(t')>0} - P_0(t) = 0$ or $P^{+}(t)|_{\Delta v(t')<0} - P_0(t) = 0$,
we have $n^{r+}_{\Delta v+} n^{r-}_{\Delta v-} - n^{r+}_{\Delta v-} n^{r-}_{\Delta v+} = 0$.
Therefore, $P^{+}(t)|_{\Delta v(t')>0} = P^{+}(t)|_{\Delta v(t')<0} = P_0(t)$.

If $P^{+}(t)|_{\Delta v(t')>0} - P_0(t) \neq 0$, we have

$$[P^{+}(t)|_{\Delta v(t')>0} - P_0(t)] \cdot [P^{+}(t)|_{\Delta v(t')<0} - P_0(t)]$$

$$= \frac{-(n^{r+}_{\Delta v-} n^{r-}_{\Delta v+} - n^{r+}_{\Delta v+} n^{r-}_{\Delta v-})^2}{(n^{r+}_{\Delta v+} + n^{r-}_{\Delta v+})(n^{r+}_{\Delta v-} + n^{r-}_{\Delta v-})(n^{r+}_{\Delta v+} + n^{r+}_{\Delta v-} + n^{r-}_{\Delta v+} + n^{r-}_{\Delta v-})^2} < 0.$$

Therefore,
if $P^{+}(t)|_{\Delta v(t')>0} - P_0(t) > 0$, we have $P^{+}(t)|_{\Delta v(t')<0} - P_0(t) < 0$;
if $P^{+}(t)|_{\Delta v(t')>0} - P_0(t) < 0$, we have $P^{+}(t)|_{\Delta v(t')<0} - P_0(t) > 0$.
